# Investigation of Anti-Dermatophytic Effects of Non-Steroidal Anti-Inflammatory Drugs on *Trichophyton* *Mentagrophytes* and *Epidermophyton* *Floccosum*

**Published:** 2011

**Authors:** Ali Abdul Hussein, S AL-Janabi

**Affiliations:** Department of Clinical Laboratory, College of Pharmacy, University of Karbala, Iraq.

**Keywords:** NSAID, Dermatophytes, *Trichophyton mentagrophytes*, *Epidermophyton floccosum*

## Abstract

Non-steroidal anti-inflammatory drugs (NSAIDs) are the most common pharmacological group that has three primary therapeutic effects including anti-inflammatory, anti-pyrexia, and analgesia. In this study, seven of NSAIDs were tested against two species of skin pathogenic fungi (dermatophytes). Percentage inhibition was determined for effective agents. Diclofenac, Aspirin and Naproxen showed much more ability to inhibit dermatophytes growth. *Epidermophyton floccosum *revealed susceptibility to more tested agents than those of *Trichophyton mentagrophytes*.

In conclusion, many of NSAIDs may have the ability to inhibit pathogenic fungi. Others may also have potential activity toward fungal growth.

## Introduction

Non-steroidal anti-inflammatory drugs (NSAIDs) are the centerpiece of pharmacotherapy for most rheumatological disorders and are used in large numbers as analgesic and antipyretics (1). In addition to these pharmaceutical functions, NSAIDs contain many agents within their group among which some possess antimicrobial activities. Exposure of fungi (which is considered an important group of eukaryotic to NSAID agents) may prevent entire fungal development or inhibit one or more stages of their life cycle. Seven of nine NSAIDs in concentration of 1 mM showed ability to inhibit biofilm formation of three strains of *Candida albicans *(2). Furthermore, cultivation of *Trichoderma viride *on media containing acetylsalicylic acid (Aspirin) or Diclofenac revealed strong and dose-dependent inhibition of colonies growth (3). Sulindac in the form of sulindac sulfide that has prophylactic effects in the prevention of colon cancer was significantly prevented *Aspergillus nidulans *to grow after exposures to 350 μM of this agent (4). Herman and Herman (5) demonstrated that Aspirin and Indomethacin inhibited growth and morphological changes of three species of fungi related to the family Saprolegniaceae. The completion of *Dipodascopsis *fungus life cycle was also inhibited by Aspirin (6). 

Dermatophytes are considered as an important group of pathogenic fungi which can infect human skin and cause lesion in cutaneous layer (7). Dermatophyte species can be classified into three genera within the fungi imperfecti, namely *Trichophyton, Microsporum*, and *Epidermophyton *(8). Keratinous materials are main source for dermatophytes nutrition. Thus, they infected skin, nail and hair. In previous study, ibuprofen as one of NSAIDs found to have therapeutic activity against dermatophytes and to treat their disease (dermatophytoses) after applied *in-vitro *and *in-vivo *(9).

Investigation for antifungal effects of NSAIDs on dermatophytes was the aim of this present study with determination of biological concentrations of any effective agents.

## Experimental

*Organisms*

Two strains of dermatophytes were clinically isolated from AL-Hussein general hospital of Karbala province in February 2009. Skin scales of fungal lesion were cultured on Sabouraud’s glucose agar of the following components: Glucose 20 g, peptone 10 g, agar 15 g, chloramphenicol 0.05 g and 1000 mL of distilled water. Cultures were incubated at 28^º^C for two weeks. Grown fungi were diagnosed according to criteria recorded by Rippon (10) and Emmons (11).

The isolated strains were *Trichophyton mentagrophytes *and *Epidermophyton floccosum*.

*Chemical agents*

Diclofenac sodium and Meloxicam were supplied from Ajanta Pharma Limited (Paithan, Maharashtra, India). Naproxen was supplied from Medical Bahri Company, (Damascus (AI-Gassoulaeh), Syria). Piroxicam was supplied from Shaghai Pharmaceutical Co. LTD, (Shaphar, China). Celecoxib was supplied from Micro-Labs, Limited, Sipcot Industrial Complex (Hosur, India). Mefenamic acid was supplied from Brown and Burk Pharmaceutical Limited, Spicot, (Hosur, India). Aspirin was supplied from SDI, (Samara, Iraq).

*Antifungal assay*

The colony diameter method recorded by Kücüc and Kivan (12) was used. Various concentrations of NSAID agents were mingled with melting prepared Sabouraud’s glucose agar containing 0.5% dimethyl sulfoxid (DMSO) for increasing NSAIDs dissolution. Then, they were poured in sterile Petri dishes. A disk (9 mm) of old grown fungi (at 28°C for 1 week) was inoculated on the center of culture media. Plates were incubated at 28^°^C for 1 week. Perpendicular colony diameters (mm) of grown strains were measured and percentage inhibition calculated according the formula: 

$\mathrm{Percentage inhibition}=\frac{(C-T)\times 100}{C}$

Where: C = colony diameter (mm) of the control; T = colony diameter (mm) of the test plate.

*Minimal inhibitory concentrations determination (MIC)*

MIC was performed according to Santos and Hamdan (13). Serial twofold dilutions of tested NSAIDs were prepared in Sabouraud’s glucose broth. The fungal colony grown at 28°C for 1 week was covered with 5 mL of sterile saline (0.9%), and the suspensions were made by gently probing the surface with the tip of Pasteur pipette. Inoculum quantification was made by counting fungal cells in a hemocytometer to obtain 2×10^4^ cfu/mL. 

Microdilution plates (96 wells) were set up in accordance with the NCCLS reference method (14). Each microdilution well containing 100 μL of the twofold drug concentration was inoculated with 100 μL of the diluted inoculum suspension. For each test plate, two drug-free controls were included; one with the medium alone and the other with 100 μL of medium plus 100 μL of inoculum suspension. The microdilution plates were incubated at 28^°^C and were read visually after 7 days of incubation.

## Results and Discussion

**Figure 1:**
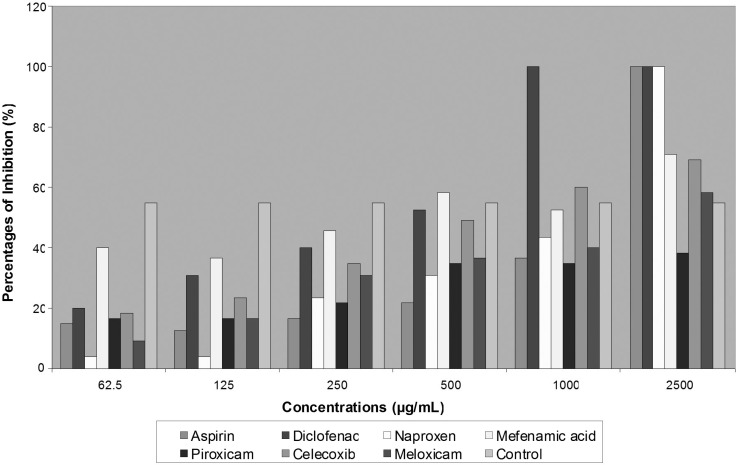
*Percentage inhibition of NSAIDs Effects on *T. mentagrophytes

Most common prescribed NSAIDs were tested for antifungal effects on two species of dermatophytes. Three of seven agents that include Diclofenac, Aspirin and Naproxen showed completely inhibition activity on fungal growth. Diclofenac revealed greatest effects on two isolated strains of dermatophytes than other agents at MIC 700 μg/mL for *T. mentagrophytes *and at 280 μg/mL for *E. floccosum*. Aspirin and Naproxen also ceased fungal development through decreased colonies diameter of growing fungi, but less than Diclofenac (Table 1). More than 50% of *T. mentagrophytes *inhibition was exhibited by Celecoxib in concentration of 1000 μg/mL and by Meloxicam in concentration of 500 μg/mL (Figure 1).

**Table 1:** MICs (μg/mL) of NSAIDs in isolated strains of dermatophytes

**NSAIDs**	***T. mentagrophytes***	***E. floccosum***
Diclofenac	700	280
Aspirin	1900	1200
Naproxen	2200	800
Mefenamic acid	< 2500	260
Piroxicam	< 2500	520
Celecoxib	< 2500	1200
Meloxicam	< 2500	1300

**Figure 2:**
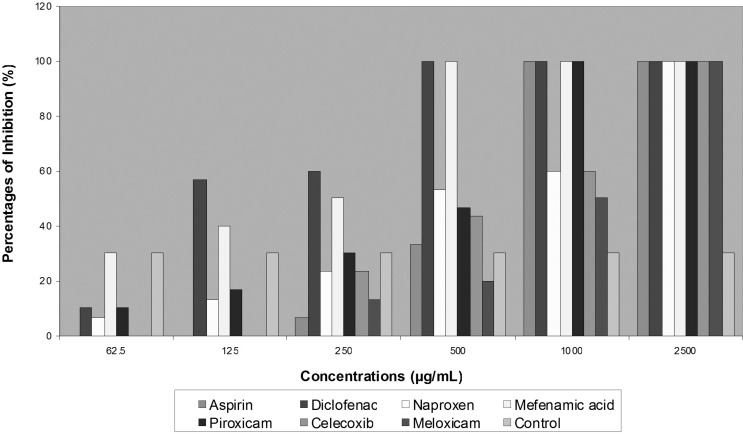
*Percentage inhibition of NSAIDs Effects on *E. floccosum

Although no activity was noted with Piroxicam on *T. mentagrophytes *and *E. floccosum *growth, they were completely inhibited in the presence of 1000 μg/mL of Piroxicam. In general, *E. floccosum *exhibited more susceptibility to all tested NSAIDs in concentration of 2500 μg/mL (Figure 2).

Since the introduction of acetylsalicylic acid (Aspirin) as the first NSAID in 1897 (15), NSAIDs have been used for years for management of pain and inflammation with good efficiency. Now, not only are they the most widely prescribed classes of medications in the world, but also remain the principle pharmacological agents for symptom relief in the patients with rheumatic diseases (16).

Susceptibility of two strains of dermatophytes to Diclofenac and Aspirin tend to indicate the presence of a potential inhibitory action by these agents on tested fungi. Antimicrobial action of Aspirin may relate to the presence of phenol group within its structure (17). However, not all NSAIDs have the same inhibitory effects on fungi. Although *Candida albicans *did not influence by Mefenamic acid, Meloxicam or Naproxen (18), Aspirin and Diclofenac showed greatest effects on this yeast through inhibition of its biofilm formation. Meanwhile, the same effects were noted with Celecoxib and Meloxicam, but to a less extent (2).

Fungi can tolerate NSAIDs and transform others to their metabolites without being influenced by these agents, such as transforming Meloxicam by 6 species of fungi (19) and Diclofenac by *Cunninghamella elegans *(20) to their metabolites through enzyme system similar to that of present in mammalian (21).

Naproxen is a Proprionic acid derivative related to the Arylacetic acid group of NSAIDs (1). Dermatophytes growth in our study was also ceased by this agent. It also has inhibitory activities on several types of organisms. Plant seeds germination was inhibited in the presence of Naproxen (22). Furthermore, Piroxicam which is a member of the oxicam group of NSAIDs (1) that have less ability to inhibit *Trichoderma viride *(3), showed the capacity of preventing persistence of *E. floccosum*.

The primary mechanism of the action of all NSAIDs in mammalians is the inhibition of cyclooxygenase (COX), a hemeprotein that exists in two isoforms (COX-1 and COX-2), and converts arachidonic acid to prostanoids such as prostaglandin (23). NSAIDs can be classified into two groups based on COX inhibition; Nonselective COX inhibitors such as Diclofenac, Naproxen and ibuprofen that have balanced inhibiting towards both COX isoforms and selective COX-2 inhibitor that inhibit COX-2 more potently than COX-1 such as Celecoxib and Meloxicam (24). However, Diclofenac and Aspirin have other effects on other enzymes, including increase catalase activity and decrease glutation peroxidase activity (25). 

COX-1 is important in the protection of human gastrointestinal mucosa. Inhibition of this enzyme can result in formation of ulcers. On the other hand, stimulation of COX-2 results in pain and inflammation (26). Therefore, inhibition of COX-2 will limit these symptoms. Although ulceration considers harmful side effects of Aspirin through inhibition of COX-1 (27), this phenomenon may be useful for making successful experimental studies that need producing fungal infection in stomach (28). Furthermore, NSAIDs, especially Aspirin may prevent gastric cancer (29) due to suppression of COX-2 (30). 

In addition to mammalian, COX can be found in several species of fungi. *Dipodascopsis uninucleata *fungus can use its COX to incorporate arachidonic acid within lipids synthesis (31). Cells of *Aspergillus *were found to have three similar in sequence genes to specify mammalian prostaglandin synthases (COX) 

(32). Prostaglandin, the product of COX activity on arachidonic acid, has been demonstrated to produce by many fungi. *Candida albicans *and *Cryptococcus neoformans *can secrete prostaglandins *de nova *or via conversion of exogenous arachidonic acid (33). Thus, treatment with COX inhibitors dramatically reduced the viability of the fungi and the productibility of prostaglandins. Synthesis of prostaglandin by biofilms and suspended cells of *C. albicans *proved to be sensitive to the COX inhibitors (Aspirin and Diclofenac) (34), otherwise, Indomethacin and Piroxicam have also capability of inhibiting prostaglandin production from *C. albicans *and *C. neoformans *(33).

Dermatophytes, the main subject of this study, also have the ability to produce prostaglandin that responsible for chronic fungal colonization (35). Therefore, activity of some tested NSAIDs on isolated dermatophytes may related to inhibitory action of these agents on secreted COX that produced by fungal cells and not to direct toxicity that NSAIDs may have. Inhibition of prostaglandin production is not the only mechanism of NSAIDs in fungal cells. Others are also recorded which include either cell cycle delay as noted in *Aspergillus nidulans *(4) or function inhibition of fungal pathogenic substances such as function inhibition of the metalloproteinases by Diclofenac and Piroxicam (36).

The usage of NSAIDs at therapeutic doses could cause many adverse effects. Some of NSAIDs cause less damage in human than others, especially Celecoxib or Rofecoxib (23) and coated Aspirin (37). However, the side effects of NSAIDs are correlated with their ability to inhibit COX-1, while inhibited COX-2 leads to anti-inflammatory (therapeutic) effects of these agents (27). For treatment of dermatophytic infections, topical drugs are beneficial. Thus, preparation of topical formulations of NSAID may become useful. Diclofenac was prepared in gel formula when mixing 3% Diclofenac with 2.5% hyaluronan gel (38). This preparation may decrease the adverse effects of NSAIDs, especially on hepatocytes (39) and cardiovascular systems (16). Therefore, successful application of NSAIDs as antimicrobial agents, especially against dermatophytes, may reduce the adverse effects of such drugs.

In conclusion, NSAIDs contain many promising agents within their group which can be used against dermatophytes. Diclofenac, Aspirin and Naproxen are more effective on two main species of dermatophytes. Other NSAID agents have variable actions on dermatophytes, based on fungal species.
